# Cardiac Complications in Women With Hypertensive Disorders of Pregnancy Undergoing a Cesarean Section: Role of Intraoperative Hemodynamic Monitoring

**DOI:** 10.7759/cureus.97135

**Published:** 2025-11-18

**Authors:** Wajeeha Fatima, Azfar Rahim, Zainab Nisar, Izza Mahoor, Ghulam Sughra, Warda Raja, Jawad Hameed, Muhammad Ammar Hasan, Muhammad Anas Ahmed, Jassim Zaheen Shah

**Affiliations:** 1 Obstetrics and Gynaecology, Mid City Hospital, Lahore, PAK; 2 Obstetrics and Gynaecology, Sindh Institute of Child Health and Neonatology, Karachi, PAK; 3 Anaesthesia, Avicenna Medical College, Lahore, PAK; 4 Obstetrics and Gynaecology, Asian Institute of Medical Sciences, Hyderabad, PAK; 5 Department of Pediatrics and Child Health, Aga Khan University Hospital, Karachi, PAK; 6 Anesthesia and Critical Care, Lady Reading Hospital, Peshawar, PAK; 7 Obstetrics and Gynaecology, Letterkenny University Hospital, Letterkenny, IRL; 8 Medicine, Shaikh Zayed Hospital, Lahore, PAK; 9 Cardiology, Heart Hospital, Doha, QAT

**Keywords:** cardiac complications, cesarean section, hemodynamic monitoring, hypertensive disorders of pregnancy, maternal outcomes

## Abstract

Introduction

Hypertensive disorders of pregnancy (HDP) are major contributors to maternal morbidity and mortality, predisposing to significant cardiac complications during cesarean section. Hemodynamic fluctuations under anesthesia further increase the risk of myocardial ischemia, arrhythmia, and pulmonary edema. Continuous intraoperative monitoring may help identify early hemodynamic instability and improve maternal outcomes.

Objective

This study aimed to evaluate the frequency, pattern, and predictors of maternal cardiac complications in women with HDP undergoing cesarean section and to determine the protective role of intraoperative hemodynamic monitoring.

Methods

This retrospective study was conducted at Lady Reading Hospital MTI, Peshawar, Pakistan, over 24 months (January 2023-December 2024). Medical records of 250 women aged 18-45 years with HDP undergoing cesarean section were reviewed. Data included demographic features, disease subtype, anesthesia type, intraoperative hemodynamic parameters, monitoring modality, and postoperative outcomes. Maternal cardiac complications were defined as arrhythmia, pulmonary edema, myocardial ischemia, heart failure, or hemodynamic instability occurring intraoperatively or within 48 hours postoperatively. Data were analyzed using IBM SPSS Statistics for Windows, version 26.0. Independent t-tests and Chi-square tests were applied, and logistic regression identified independent predictors (p < 0.05 considered significant).

Results

Among 250 women, preeclampsia was the most common subtype (162; 64.8%), followed by eclampsia (48; 19.2%) and gestational hypertension (11.2%). Overall, 54 (21.6%) developed maternal cardiac complications. Advanced maternal age (≥35 years), elevated body mass index (BMI) (≥28 kg/m²), and general anesthesia were independently associated with increased cardiac risk (p < 0.05). Invasive intraoperative hemodynamic monitoring significantly reduced the likelihood of undetected hemodynamic deterioration (adjusted odds ratio (OR) 0.48; 95% confidence interval (CI) 0.25-0.94; p = 0.031). Patients with complications had longer hospital stays and higher ICU admission rates.

Conclusion

Maternal cardiac complications are common in women with HDP undergoing a cesarean section, especially among older and obese patients and those receiving general anesthesia. Invasive hemodynamic monitoring provides a protective benefit and should be considered in high-risk patients to enhance perioperative safety and improve outcomes.

## Introduction

Pulmonary hypertension is defined as an increase in the mean pulmonary arterial pressure of more than 25 mmHg at rest [1] or more than 30 mmHg with exercise, as measured by echocardiography or right heart catheterization [2]. It involves a progression of pulmonary vascular remodeling, narrowing of vessels, and in situ thrombosis, which ultimately leads to progressive right ventricular failure. The etiological factors may be idiopathic or secondary to other underlying conditions such as collagen vascular diseases, congenital systemic shunts, human immunodeficiency virus (HIV) infection, or medication use. However, the most common secondary causes include left heart diseases, chronic lung diseases such as chronic obstructive pulmonary disease (COPD), and chronic thromboembolic pulmonary hypertension [3,4]. Pulmonary hypertension and other cardiac complications that develop during pregnancy contribute substantially to maternal morbidity and mortality worldwide [3].

In women with hypertensive disorders of pregnancy (HDP), including preeclampsia, eclampsia, and chronic hypertension with superimposed preeclampsia, the risk of cardiac complications is markedly increased due to elevated systemic vascular resistance, endothelial dysfunction, and impaired myocardial relaxation [4]. These pathological alterations predispose affected women to heart failure, pulmonary edema, arrhythmias, and myocardial ischemia during the peripartum period, particularly under the physiological and anesthetic stress of cesarean delivery. Performing cesarean section in such high-risk patients presents unique hemodynamic challenges that necessitate vigilant intraoperative monitoring and carefully titrated anesthesia to maintain cardiovascular stability [2].

Anesthetic agents and techniques exert profound effects on maternal cardiovascular performance [5]. Agents causing pulmonary vasoconstriction, such as nitrous oxide, should be avoided in women with pulmonary hypertension due to their potential to exacerbate right ventricular strain [6]. Both general and regional anesthesia can influence cardiac output through vasodilation, reduced venous return, and diminished coronary perfusion, increasing the risk of hemodynamic instability [5]. Structural cardiac abnormalities, particularly valvular lesions such as severe mitral regurgitation and moderate tricuspid regurgitation, are commonly encountered in pregnancy and may further complicate perioperative management [7]. The anesthetic goals in such patients include avoiding increases in systemic vascular resistance, maintaining sinus rhythm, preventing myocardial depression, and ensuring adequate preload and venous return. Controlled regional anesthesia may offer hemodynamic advantages by lowering afterload and enhancing cardiac output when cautiously titrated [8].

Given the hemodynamic lability inherent to HDP, continuous intraoperative hemodynamic monitoring plays a crucial role in guiding fluid therapy, anesthetic depth, and vasopressor administration. Hemodynamic instability in this context is generally defined as sustained systolic blood pressure below 90 mmHg or a decrease of more than 20% from baseline, mean arterial pressure below 65 mmHg, or heart rate exceeding 120 beats per minute, requiring pharmacological or fluid intervention [3]. Continuous invasive arterial pressure and cardiac output monitoring facilitate early detection and prompt correction of such disturbances, thereby reducing the incidence of serious cardiac events during cesarean delivery. Previous studies have demonstrated that early recognition of hemodynamic deterioration through invasive monitoring significantly lowers maternal morbidity and improves perioperative outcomes in high-risk obstetric populations [3].

Despite improvements in obstetric and anesthetic management, maternal cardiac complications remain a leading cause of morbidity and mortality in women with hypertensive disorders of pregnancy undergoing a cesarean section. Therefore, the present study aims to determine the frequency, types, and predictors of maternal cardiac complications in this population and to assess the role of intraoperative invasive hemodynamic monitoring in improving perioperative outcomes.

## Materials and methods

This retrospective study was conducted in the Department of Obstetrics and Gynecology and Department of Anesthesiology at Lady Reading Hospital, MTI Peshawar, Pakistan, over a period of 24 months, from January 2023 to December 2024. As this was a retrospective analysis based entirely on hospital records without any direct patient interaction or intervention, ethical approval was not required. However, the study was reviewed by the institutional review board for approval to retrieve the data. All data were anonymized to ensure patient confidentiality. The approval number is 169/LHR/MTI, dated 01/01/2023.

The study population included women aged 18-45 years who were diagnosed with hypertensive disorders of pregnancy, specifically gestational hypertension, preeclampsia, eclampsia, or chronic hypertension with superimposed preeclampsia, and who underwent a cesarean section under either spinal or general anesthesia at the study center. The sample size was calculated using OpenEpi version 3.01 (Atlanta, GA, USA), assuming a prevalence of maternal cardiac complications of 20% among hypertensive parturients undergoing cesarean section, with a 95% confidence interval (CI) and a 5% margin of error. The required sample size was 246, rounded to 250 to account for incomplete records. A non-probability consecutive sampling technique was used to include all eligible cases within the study period.

Inclusion criteria consisted of all singleton pregnancies complicated by hypertensive disorders, gestational age ≥28 weeks, and availability of complete intraoperative and postoperative monitoring data. Exclusion criteria included patients with pre-existing cardiac disease, multiple gestations, significant thyroid or renal dysfunction unrelated to pregnancy, and incomplete or missing anesthetic or hemodynamic records.

Data were retrieved from patient files, anesthesia charts, and postoperative recovery records. Collected parameters included demographic details (age, parity, body mass index (BMI), and gestational age), obstetric data (type of hypertension and urgency of cesarean section), and preoperative investigations (hemoglobin, serum electrolytes, liver enzymes, and renal function tests). Baseline systolic, diastolic, and mean arterial pressures, along with resting heart rate, were recorded before induction or spinal anesthesia.

Intraoperative hemodynamic monitoring data were obtained from anesthetic charts. All patients were monitored using standard American Society of Anesthesiologists (ASA) monitoring, including non-invasive blood pressure (NIBP), electrocardiogram (ECG), pulse oximetry, and end-tidal carbon dioxide (EtCO₂). In patients with severe preeclampsia or anticipated hemodynamic instability, invasive arterial pressure and central venous pressure (CVP) monitoring were utilized. Blood pressure and heart rate were recorded at 5-minute intervals throughout the surgery.

Maternal cardiac complications were defined as any of the following occurring intraoperatively or within 48 hours postoperatively: arrhythmia detected on ECG, myocardial ischemia indicated by ECG changes or elevated cardiac troponin, pulmonary edema confirmed by clinical and radiological findings, acute heart failure assessed clinically and by echocardiography, or significant hemodynamic instability requiring vasopressor support. All suspected cardiac events were reviewed and confirmed by a consultant anesthesiologist and cardiologist.

Intraoperative details such as type of anesthesia, fluid therapy (total volume administered), estimated blood loss, urine output, duration of surgery, and use of vasopressors (type and total dose) were recorded. Postoperative outcomes, including the occurrence of cardiac complications, need for ICU admission, and length of hospital stay, were also documented.

Data were entered and analyzed using IBM SPSS Statistics for Windows, version 26.0 (released 2018, IBM Corp., Armonk, NY). Continuous variables, including age, BMI, gestational age, baseline mean arterial pressure, intraoperative mean arterial pressure, and heart rate, were expressed as mean ± standard deviation and compared between groups (with and without cardiac complications) using independent sample t-tests. Categorical variables such as type of hypertension, type of anesthesia, presence of invasive monitoring, and occurrence of cardiac complications were presented as frequencies and percentages and analyzed using the Chi-square test. Logistic regression analysis was performed to identify independent predictors of maternal cardiac complications, adjusting for confounding factors such as maternal age, baseline mean arterial pressure, and type of anesthesia. A p-value of less than 0.05 was considered statistically significant.

All data were handled with strict confidentiality. Patient identifiers were removed prior to analysis, and findings were used solely for research and quality improvement purposes.

## Results

Maternal demographics and baseline clinical characteristics

A total of 250 women with HDP who underwent cesarean section were included in this retrospective analysis. The mean age of participants was 30.8 ± 5.2 years, and the mean gestational age was 35.6 ± 2.8 weeks. Among the total, 162 (64.8%) had preeclampsia, 48 (19.2%) had eclampsia, 28 (11.2%) had gestational hypertension, and 12 (4.8%) had chronic hypertension with superimposed preeclampsia. Overall, 54 (21.6%) women developed maternal cardiac complications either intraoperatively or within 48 hours postoperatively.

Table 1 presents the baseline demographic and clinical characteristics. Women who developed cardiac complications were significantly older (32.7 ± 4.9 years) compared to those without complications (30.3 ± 5.1 years, p = 0.003). The mean BMI was also higher in the complication group (29.4 ± 3.8 kg/m²) than in the non-complication group (27.1 ± 3.6 kg/m², p < 0.001). Baseline systolic and diastolic blood pressures were significantly elevated among those who experienced cardiac events.

**Table 1:** Baseline demographic and clinical characteristics of study participants (n = 250) Values are presented as mean ± standard deviation. *Significant at p < 0.05.

Variable	Total (n = 250)	With cardiac complications (n = 54, 21.6%)	Without complications (n = 196, 78.4%)	p-value
Age (years)	30.8 ± 5.2	32.7 ± 4.9	30.3 ± 5.1	0.003*
BMI (kg/m²)	27.6 ± 3.8	29.4 ± 3.8	27.1 ± 3.6	<0.001*
Parity	2.2 ± 1.1	2.3 ± 1.2	2.1 ± 1.0	0.274
Gestational age (weeks)	35.6 ± 2.8	35.1 ± 2.9	35.8 ± 2.7	0.187
Systolic blood pressure (BP) (mmHg)	158.2 ± 18.4	166.8 ± 20.2	156.0 ± 17.3	0.001*
Diastolic BP (mmHg)	99.6 ± 10.3	104.3 ± 9.8	98.3 ± 10.1	0.002*

Distribution of hypertensive disorders and type of anesthesia

As shown in Table 2, preeclampsia (162; 64.8%) was the most common hypertensive disorder. Cardiac complications were most frequent among women with eclampsia (16; 33.3%) and chronic hypertension with superimposed preeclampsia (5; 41.7%). Regarding anesthesia, 182 (72.8%) underwent spinal anesthesia, and 68 (27.2%) underwent general anesthesia. The incidence of cardiac complications was significantly higher in those who received general anesthesia (24; 35.3%) than in those who received spinal anesthesia (30; 16.5%, p = 0.002).

**Table 2:** Type of hypertensive disorder and anesthesia in relation to cardiac complications (n = 250) Values are presented as n (%). *Significant at p < 0.05.

Variable	Total	With cardiac complications	Without complications	p-value
Type of hypertensive disorder				
Gestational hypertension	28 (11.2%)	2 (7.1%)	26 (92.8%)	0.041*
Preeclampsia	162 (64.8%)	31 (19.1%)	131 (80.8%)	0.192
Eclampsia	48 (19.2%)	16 (33.3%)	32 (66.6%)	0.036*
Chronic HTN + preeclampsia	12 (4.8%)	5 (41.7%)	7 (58.3%)	0.044*
Type of anesthesia				
Spinal	182 (72.8%)	30 (16.5%)	152 (83.5%)	0.002*
General	68 (27.2%)	24 (35.3%)	44 (64.7%)	0.032

Intraoperative hemodynamic parameters and monitoring techniques

Invasive monitoring was utilized in 76 (30.4%) cases, primarily those with severe preeclampsia or eclampsia. The criteria used for invasive monitoring lost about half of the cases with cardiac complications. Cardiac complications were more frequent among patients monitored invasively (26; 48.1%) compared to those with standard non-invasive monitoring (28; 16.1%, p = 0.009). Patients who developed cardiac complications had significantly higher mean intraoperative systolic, diastolic, and mean arterial pressures as well as higher mean heart rate (Table 3).

**Table 3:** Intraoperative hemodynamic monitoring and parameters (n = 250) *Significant at p < 0.05.

Parameter	Total n (%) / Mean ± SD	With cardiac complications (n = 54, 21.6%)	Without complications (n = 196, 78.4%)	p-value
Invasive monitoring used	76 (30.4%)	26 (48.1%)	50 (25.5%)	0.009*
Mean systolic BP (mmHg)	142.6 ± 16.5	154.3 ± 18.2	139.4 ± 15.3	<0.001*
Mean diastolic BP (mmHg)	88.3 ± 10.4	94.2 ± 9.7	86.8 ± 10.1	<0.001*
Mean heart rate (bpm)	99.1 ± 11.6	106.8 ± 12.3	97.1 ± 10.7	<0.001*
Intraoperative hypotension episodes	64 (25.6%)	21 (38.9%)	43 (21.9%)	0.018*
Vasopressor use	92 (36.8%)	28 (51.9%)	64 (32.7%)	0.014*

Types and frequency of cardiac complications

The types and frequencies of cardiac complications are detailed in Table 4. Arrhythmia (19; 35.2%) was the most frequent event, followed by hemodynamic instability (16; 29.6%), pulmonary edema (9; 16.7%), myocardial ischemia (6; 11.1%), and acute heart failure (4; 7.4%).

**Table 4:** Frequency and types of maternal cardiac complications (n = 54)

Complication	n (%)
Arrhythmia	19 (35.2%)
Hemodynamic instability	16 (29.6%)
Pulmonary edema	9 (16.7%)
Myocardial ischemia	6 (11.1%)
Acute heart failure	4 (7.4%)

Postoperative outcomes

Postoperative outcomes are presented in Table 5. The mean hospital stay was significantly longer in patients with cardiac complications (6.4 ± 2.1 days) compared to those without (3.2 ± 1.3 days, p < 0.001). ICU admission was required for 18 (33.3%) patients with cardiac complications versus nine (4.6%) without (p < 0.001). Similarly, 11 (20.4%) patients with complications required postoperative vasopressor support compared to eight (4.1%) in the non-complication group (p = 0.002). Maternal mortality occurred in 2 (3.7%) cases with cardiac complications.

**Table 5:** Postoperative outcomes of study participants (n = 250) *Significant at p < 0.05.

Variable	With cardiac complications (n = 54, 21.6%)	Without complications (n = 196, 78.4%)	p-value
ICU admission n (%)	18 (33.3%)	9 (4.6%)	<0.001*
Hospital stay (days, mean ± SD)	6.4 ± 2.1	3.2 ± 1.3	<0.001*
Postoperative vasopressor support n (%)	11 (20.4%)	8 (42%)	0.002*
Maternal mortality n (%)	2 (3.7%)	0 (0%)	0.041*

Predictors of maternal cardiac complications

Binary logistic regression analysis was performed to identify independent predictors of maternal cardiac complications among women with hypertensive disorders of pregnancy undergoing caesarean section (Table 6). After adjusting for confounding variables, maternal age ≥35 years (adjusted OR = 2.31; 95% CI = 1.08-4.95; p = 0.031), BMI ≥28 kg/m² (adjusted OR = 2.74; 95% CI = 1.34-5.61; p = 0.006), and the use of general anaesthesia (adjusted OR = 2.89; 95% CI = 1.42-5.87; p = 0.003) were identified as significant predictors of cardiac complications. Conversely, the use of intraoperative invasive hemodynamic monitoring had a significant protective effect, reducing the likelihood of undetected hemodynamic instability (adjusted OR = 0.48; 95% CI = 0.25-0.94; p = 0.031).

**Table 6:** Binary logistic regression analysis of predictors of maternal cardiac complications (n = 250) OR: odds ratio; CI: confidence interval; BMI: body mass index

Predictor variable	Unadjusted OR (95% CI)	p-value	Adjusted OR (95% CI)	p-value
Maternal age ≥35 years	2.08 (1.05–4.12)	0.036	2.31 (1.08–4.95)	0.031
BMI ≥28 kg/m²	2.56 (1.30–5.03)	0.007	2.74 (1.34–5.61)	0.006
Severe preeclampsia	1.94 (0.98–3.83)	0.057	1.82 (0.87–3.79)	0.108
Use of general anesthesia	2.75 (1.38–5.50)	0.004	2.89 (1.42–5.87)	0.003
Duration of surgery >90 min	1.68 (0.85–3.31)	0.136	1.51 (0.72–3.16)	0.274
Invasive hemodynamic monitoring	0.52 (0.27–0.98)	0.042	0.48 (0.25–0.94)	0.031

## Discussion

This retrospective study evaluated 250 women with HDP who underwent cesarean section and found a notable incidence of maternal cardiac complications (21.6%). The findings highlight the complex interplay between maternal demographics, disease subtype, anesthesia type, and intraoperative hemodynamic management. Consistent with previous studies, our results emphasize that advanced maternal age, obesity, and use of general anesthesia increase the likelihood of cardiac complications, whereas invasive intraoperative monitoring may provide a protective benefit by enabling timely hemodynamic intervention.

Advanced maternal age (≥35 years) and higher BMI (≥28 kg/m²) emerged as independent predictors of cardiac complications. Increasing maternal age is associated with reduced myocardial compliance, diminished diastolic reserve, and higher rates of subclinical cardiac dysfunction, predisposing to decompensation during the hemodynamic stress of cesarean section [9,10]. Similarly, obesity increases plasma volume, cardiac workload, and systemic vascular resistance, which exacerbate left ventricular hypertrophy and diastolic dysfunction [11]. Prior cohort studies have demonstrated that obese parturients have nearly a twofold higher risk of perioperative cardiovascular instability compared with normal-weight women [12].

Baseline hypertension also contributed significantly to adverse outcomes. Elevated systolic and diastolic pressures before anesthesia induction reflect chronic vascular remodeling and endothelial dysfunction, key features of HDP that predispose to myocardial strain and left ventricular failure [13]. Although endothelial dysfunction is central to preeclampsia, our results showed that isolated preeclampsia did not independently predict cardiac complications once confounding factors were controlled. This can be explained by the protective effect of optimized intraoperative management, including invasive hemodynamic monitoring and strict blood pressure control, which were more consistently implemented in women with severe preeclampsia. By contrast, patients with eclampsia or chronic hypertension with superimposed preeclampsia, who often presented emergently and without prior optimization, exhibited significantly higher complication rates. These findings suggest that chronic vascular remodeling, rather than transient endothelial dysfunction alone, represents the principal determinant of cardiac events in HDP, as persistent structural vascular changes and diastolic stiffness predispose to decompensation under anesthetic stress.

Interestingly, gestational hypertension also demonstrated a statistical association with cardiac complications despite being a transient condition. The small number of affected cases suggests that even short-term hypertensive surges can unmask subclinical cardiac dysfunction when combined with abrupt hemodynamic shifts during anesthesia. However, the limited number of events in this subgroup warrants cautious interpretation.

The incidence of complications was highest among women with eclampsia and chronic hypertension with superimposed preeclampsia. This gradient aligns with previous observations that the chronicity and severity of hypertension directly correlate with the degree of myocardial involvement [4]. Eclampsia produces severe vasospasm, endothelial disruption, and microvascular dysfunction, leading to capillary leak, pulmonary congestion, and impaired coronary perfusion, all of which increase cardiac risk.

Anesthetic technique substantially influenced outcomes. Women undergoing cesarean section under general anesthesia experienced a higher frequency of cardiac complications compared to those receiving spinal anesthesia. These results coincide with Wang et al. [14], who demonstrated that neuraxial anesthesia provides more stable hemodynamics and shorter ICU stays in pregnant women with severe pulmonary hypertension compared with general anesthesia. General anesthesia increases myocardial oxygen demand during laryngoscopy and intubation, depresses contractility via volatile agents, and raises pulmonary vascular resistance-deleterious effects in hypertensive and volume-sensitive patients [3]. By contrast, controlled neuraxial anesthesia can reduce systemic vascular resistance gradually, preserving venous return and cardiac output when carefully titrated [15]. Nonetheless, the greater risk seen with general anesthesia may also reflect confounding by indication, as it was frequently employed in the most severe or emergent cases. Even after adjustment, general anesthesia remained an independent predictor, suggesting that both the anesthetic choice and disease severity contribute synergistically to adverse outcomes.

Invasive monitoring was employed in nearly one-third of cases, primarily in patients with severe disease. While unadjusted analyses suggested higher complication rates in this group, multivariate analysis revealed that invasive monitoring independently reduced the odds of undetected hemodynamic deterioration (adjusted OR 0.48; p = 0.031). This protective effect likely represents the benefit of early corrective interventions, such as vasopressor titration, fluid management, and anesthetic adjustment, facilitated by continuous real-time data, rather than the monitoring itself [16]. Therefore, intraoperative invasive monitoring should be considered a dynamic tool that mobilizes prompt clinical responses to evolving instability.

Intraoperative hypotension was observed in one-fourth of patients, but only about one-third of those developed cardiac complications. This suggests that hypotension acts as a precipitating factor rather than an isolated cause of cardiac events. Patients with preserved myocardial reserve tolerated transient hypotensive episodes, whereas those with preexisting diastolic dysfunction or ventricular hypertrophy were more vulnerable to decompensation. These hemodynamic fluctuations can compromise myocardial oxygen balance, trigger arrhythmogenesis, and precipitate ischemia in predisposed women.

Among those who developed complications, arrhythmia (35.2%) and hemodynamic instability requiring vasopressors (29.6%) were most frequent, followed by pulmonary edema, myocardial ischemia, and acute heart failure. This spectrum mirrors findings in other high-risk obstetric populations, where arrhythmias and pulmonary edema dominate acute cardiac presentations [7,8]. Pulmonary edema arises from the combined effects of high afterload, endothelial leak, and volume redistribution during delivery, while myocardial ischemia and heart failure, although less common, are major causes of postpartum ICU admission and mortality. Notably, our data revealed that patients with cardiac complications had markedly longer hospital stays and higher ICU admission rates. Two maternal deaths occurred, underscoring the need for proactive multidisciplinary management.

Binary logistic regression confirmed that maternal age ≥35 years, BMI ≥28 kg/m², and general anesthesia were independent predictors of cardiac complications, whereas invasive monitoring had a significant protective influence. The absence of a predictive role for severe preeclampsia likely reflects the impact of vigilant perioperative management protocols, which mitigated its effect on cardiac outcomes. These results are consistent with prior evidence demonstrating that when aggressive monitoring and hemodynamic optimization are applied, the severity of HDP alone does not independently predict cardiac morbidity [14,16].

This study underscores some important clinical implications. First, preoperative risk stratification using age, BMI, and disease chronicity is critical for anticipating intraoperative hemodynamic challenges. Second, neuraxial anesthesia should be preferred in hemodynamically stable HDP patients, provided coagulation parameters allow. Third, invasive hemodynamic monitoring should not be restricted to severe or chronic HDP but should be applied to all such patients undergoing cesarean delivery, given the unpredictable cardiovascular lability across subtypes. Finally, postoperative cardiac surveillance and early ICU admission should be considered for high-risk patients, as most cardiac complications manifest within 48 hours of delivery.

The study has several limitations. It was retrospective and single-centered, introducing potential selection bias. We lacked standardized documentation of HDP severity grading and detailed quantification of cardiac involvement (e.g., echocardiographic or biomarker data), which may have limited subgroup precision. Despite these constraints, the consistent patterns observed and alignment with existing literature enhance the reliability of our conclusions.

## Conclusions

Maternal cardiac complications are frequent among women with HDP undergoing a cesarean section, particularly in those of advanced age, elevated BMI, or severe disease requiring general anesthesia. Invasive intraoperative hemodynamic monitoring appears to mitigate the risk of unrecognized instability and subsequent cardiac morbidity. These findings advocate for individualized anesthetic planning and vigilant hemodynamic monitoring to improve maternal safety in high-risk obstetric populations.
